# Comparison of the Accuracy of Fixture-Level Implant Impression Making with Different Splinting Techniques

**DOI:** 10.1155/2021/2959055

**Published:** 2021-10-14

**Authors:** Mehrdad Nateghi, Ramin Negahdari, Sahar Molaei, Ali Barzegar, Sepideh Bohlouli

**Affiliations:** ^1^Department of Prosthodontics, Faculty of Dentistry, Tabriz University of Medical Sciences, Tabriz, Iran; ^2^Department of Oral Medicine, Faculty of Dentistry, Tabriz University of Medical Sciences, Tabriz, Iran

## Abstract

**Objectives:**

The impression-taking technique is one of the most critical factors that not only prevents the shrinkage caused by polymerization but also enhances the accuracy of implant impressions. Also, choosing the right time of taking impressions after splinting implants is one of the important criteria that affects the impression-taking technique. Accordingly, the present study aimed to evaluate the accuracy of different splint methods for implant impressions made at different times.

**Methods:**

In this in vitro study, a two-piece metallic index was prepared, and the patient's jaw was simulated by placing self-cured acrylic resin in the lower part of the index. Then, two holes were made in the acrylic resin at a specific distance from each other, and the analogs were placed in these holes. Splinting of impression copings was carried out with autopolymerized acrylic resin (GC Pattern resin LS, GC America Inc., USA), and an open tray impression approach was performed. Thirty-six casts in three groups (*n* = 12) were fabricated from the acrylic model. After scanning the casts, the impression accuracy was compared between the three study groups by measuring the distance between the outer portions of the scan bodies screw-retained on implant analogs inside the cast using the Exocad software (2015.07 version). Group 1: splinting impression copings with autopolymerized acrylic resin and impression making immediately after the setting time (4 minutes); group 2: splinting and impression procedure after 17 minutes with splint sectioning and reconnection; group 3: splinting and impression procedure after 24 hours with splint sectioning and reconnection. The data were analyzed using SPSS 17 using the Kruskal–Wallis test.

**Results:**

The mean distance measured in group 1 was 19.14 ± 0.029 mm, which was significantly lower than the main model. The distances were 19.15 ± 0.039 and 19.159 ± 0.33 mm in groups 2 and 3, respectively. These two groups were not significantly different from the main model. Moreover, the mean distance measured in the three impression techniques was similar.

**Conclusions:**

There was no significant difference in the measurements between group 2, group 3, and the main model. Therefore, dentists can make an impression after 17 minutes to reduce chair time.

## 1. Introduction

Dental implants are substitutes for conventional prostheses with favorable long-term outcomes in patients who have lost all or some of their teeth [1–4]. The success of an implant depends on the passive fit of the prosthesis and its components. Therefore, accurate impressions and the correct transfer of implant position to the cast are of high importance [5], and the clinician will receive results of an accurate impression by good adaptation of final prosthesis and reducing chair time [6]. Improper impression leads to prosthesis insertion in the wrong position, loss of osteointegration, and a prosthesis with unsatisfactory adaptation [7].

Several factors affect impression accuracy, including angulation of implant, the number of implants, polymerization shrinkage of impression material, expansion of dental stone, tray rigidity, and impression technique [8]. Correct positioning and angulation of implants has become more reliable with the use of CBCT images in primary treatment planning and purpose of analyzing hard tissues and soft tissues [9]. Moreover, MRI-based computer-assisted implant surgery is demonstrated to be an accurate procedure [10]. According to studies, misfit in the prosthesis also effects the outline and amount of stress distribution in the prosthesis and surrounding bone which may cause adverse complications. These adverse complications may include the fracture in the different sections of the implant system, framework break, or porcelain fracture, loosening of the abutment and retaining screws, marginal bone loss, and even pain or loss of osseointegration. Therefore, diminishing the misfit and improving the passive fit over variation in impression methods and impression materials is an important goal in prosthesis knowledge and dental implants [11]. Dimensional stability and polymerization shrinkage might be influenced by the impression technique (i.e., splinting or not splinting of impression copings) [12].

Numerous studies with different findings have been conducted on the idea of the “splint technique.” Some researchers agree with the idea [13, 14], while some others do not [15, 16].

Most previous studies have assumed that the splint technique during implant impression with internal connections is more accurate [17].

Autopolymerizing acrylic resin is commonly used for splinting during the open tray implant impression procedure. Mixing the powder and liquid leads to unwanted “polymerization shrinkage,” affecting the accuracy of impression and distorting implant prostheses [16].

According to Mojon et al., the highest degree of shrinkage (80%) occurs in the first 17 minutes after mixing the powder and liquid. They reported that 7.9% of shrinkage occurred in 24 hours, and measurable alterations in polymerization shrinkage did not happen after 24 hours [18]. Moreover, Gibbs et al. reported 5.72% and 5.07% volumetric shrinkage rates for GC Pattern resin and DuraLay in 20 minutes, respectively [12]. Autopolymerized acrylic resins on the market have varying shrinkage rates; for example, the shrinkage of pattern GC LS is more than DuraLay in 17 minutes [19].

In addition to time, the splint technique affects distortion and polymerization shrinkage. Sectioning and reconnecting the splint can prevent distortion and shrinkage of splint material and fracture [19]. Lee and Cho in 2011 evaluated the effect of splinting materials and methods on impression accuracy. They concluded that making an impression of splinted impression copings applying autopolymerized resin with sufficient time for polymerization (24 h) coupled with sectioning for compensating polymerization shrinkage is the most accurate splinting method [20].

The results of studies on the effect of splint impression copings during implant impressions are controversial. Due to the controversial findings of various studies concerning the effect of splinted impression copings during implant impressions, it is necessary to overcome the shrinkage caused by the polymerization and assess the impression timing after component splinting and the role of sectioning considering the availability of diverse splint techniques.

The present study aimed to determine the better impression-taking time after splinting of impression copings. The impact of sectioning of the splint was compared after 17 minutes with impression after 24 hours on impression accuracy. If there is no significant difference between the impression accuracy at different times, dentists can apply the impression technique after 17 minutes. The latter method reduces chair time and results in higher treatment success by selecting the best technique with the highest accuracy and the least defects.

## 2. Materials and Methods

### 2.1. Methods of Sample Size Determination

Sample size determination was based on pilot study's outcome. The number of samples in each group was 8, considering 0/02 difference and 0/012 standard deviation with 80% power and 5% significance. Then, we added 20% more in order to enhance the quality of study. The final number became 12.

### 2.2. Design of the Study

The present experimental in vitro study was conducted on 36 samples in three groups (*n* = 12) obtained using the open tray impression technique. The interimplant distance was digitally measured by the measurement tool in CAD software. After collecting and analyzing data, results were obtained.

### 2.3. Reference Model Fabrication

**Figure 1:**
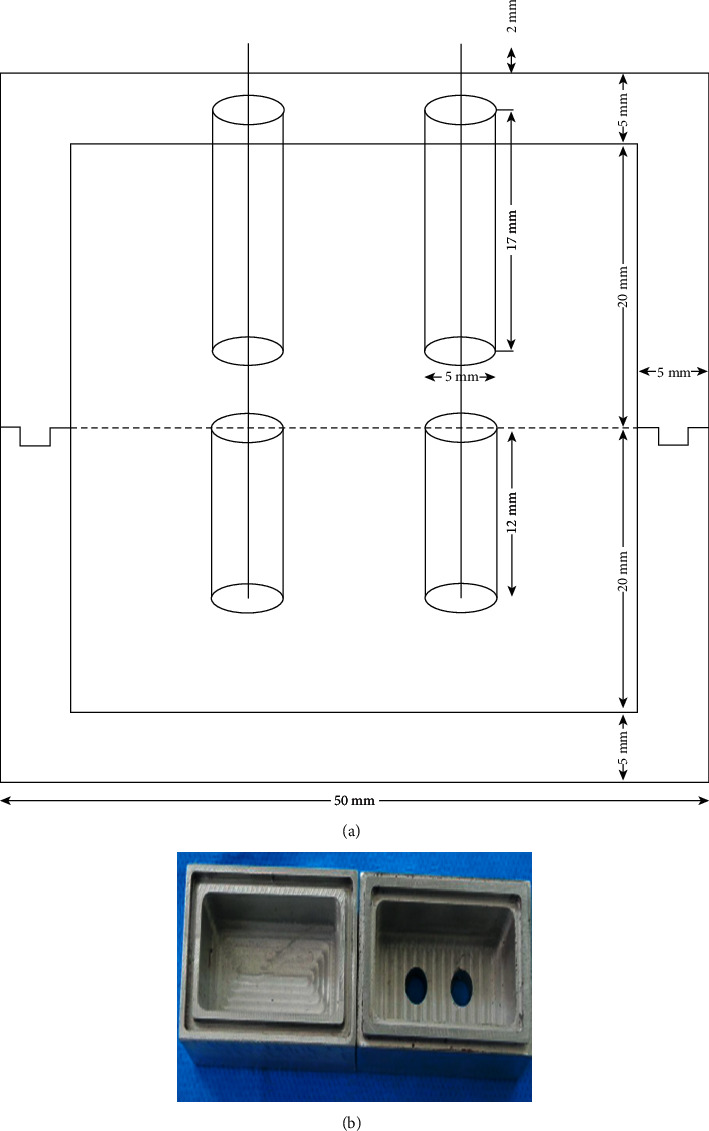
(a) Dimensions of aluminum index. (b) An aluminum index to unify the samples.

To unify the samples and perform the impression-taking process, first, an aluminum index was made (Figure 1(a)), consisting of two separate components, namely, segment A (inferior section) and segment B (superior section), which could be attached and fixed by matching parts.

Two orifices were improvised in segment B, which served as the impression tray. The orifices had diameters larger than impression copings to let them exit (Hex, Short, Dentis Corporation, South Korea). First, segment A was dipped with lubricant followed by filling to the edge of the mold with autopolymerized acrylic resin (Triplex Cold, Ivoclar Vivadent, Schaan Liechtenstein) that mimicked the lower jaw of a patient. An aluminum index to unify the samples is shown in Figure 1(b).

**Figure 2:**
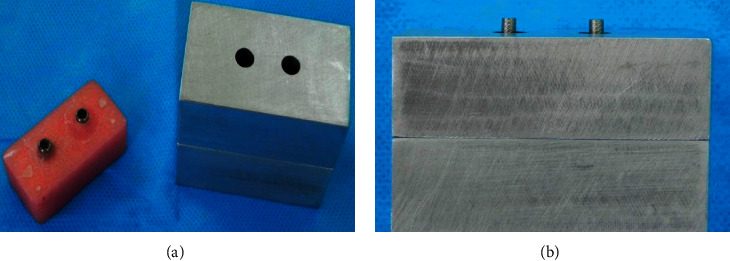
An aluminum index to unify the samples. (a) Aluminum index with two orifices with a diameter of 4.25 mm and acrylic resin model and (b) remaining two millimeters of impression copings outside the index to access the screw-retained impression copings.

Two orifices with a diameter of 4.25 mm and a depth of 12 mm were made in the acrylic resin (since 1 mm of the analog head was out of acrylic resin, we could measure the distance between the two analogs). The parallel condition of the analogs was performed on a surveyor, and the analogs were placed on a horizontal surface (Figures 2(a) and 2(b)).

Two analogs (Lab Analog Dentis Corporation, South Korea) were located in the orifices using autopolymerized resin. After complete setting, all the analogs were coded, and numbers 1 and 2 of each analog were written on the model. One polyether ether ketone (PEEK) scanbody was selected for each analog (numbers 1 and 2). These scan bodies were screw-retained on the analogs with the same numbers in a clockwise direction with a 5 Ncm torque.

Contrast spray was used for better reading, but the spray was removed from the specified parts on the scan body to eliminate error probability. The main model was scanned by the laboratory scanner (Dental Laboratory 3-Dimensional (3D) Scanner, Rainbow, Dentium) and CAD software (Dental CAD, Exocad).

### 2.4. Groups of Study

The impression coping proportionate with analogs was selected and fixed by screw-retained. Afterward, a one-step impression was taken through the open tray method in the three groups by soft putty and light body (Panasil, Kettenbach GmbH and Co., KG, Germany). Following the connection of analogs, plaster casts were prepared by type IV stone. The impression technique was conducted as follows in the three groups:

1. Impression copings were splinted with autopolymerized acrylic resin (GC Pattern resin LS, GC America Inc., USA), and the impression was taken immediately after the setting time (4 minutes).
2. Impression copings were splinted, and the impression was taken after 17 minutes by sectioning and reconnecting the splint.
3. Impression copings were splinted, and the impression was taken after 24 h by sectioning and reconnecting the splint. The time of 24 h was selected according to Mojon et al. They reported that the highest degree of shrinkage (80%) occurs in the first 17 minutes after mixing the powder and liquid. They also reported that 7.9% of shrinkage occurred in 24 hours, and measurable alterations in polymerization shrinkage did not happen after 24 hours [18].

**Figure 3:**
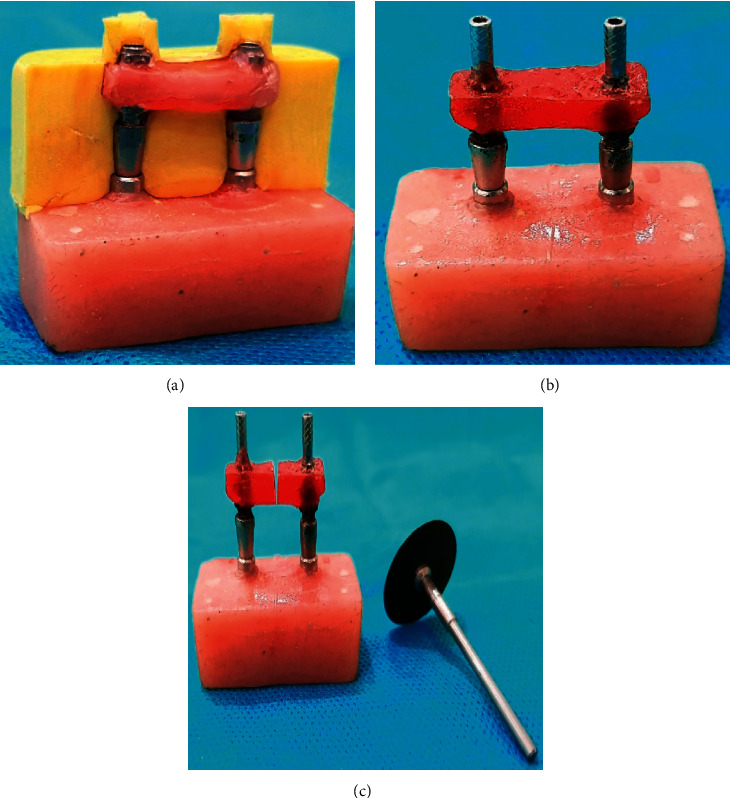
(a) Impression copings splinted with wax and the putty index prepared for controlling the volume of splinting material. (b) The impression copings splinted with autopolymerizing acrylic resin. (c) Sectioning the splinting material by using disk.

To unify the volume of splinting material in all the groups, first, impression copings were splinted by wax measuring 4 ∗ 20 ∗ 4 mm [21]. Next, a lubricant was applied on segment B, putty impression material was placed in it, and segment B was set on segment A containing splinted impression copings. The impression material was removed with impression copings, and a putty index was prepared for splinting material measuring 4 ∗ 20 ∗ 4 mm (Figures 3(a)–3(c)).

**Figure 4:**
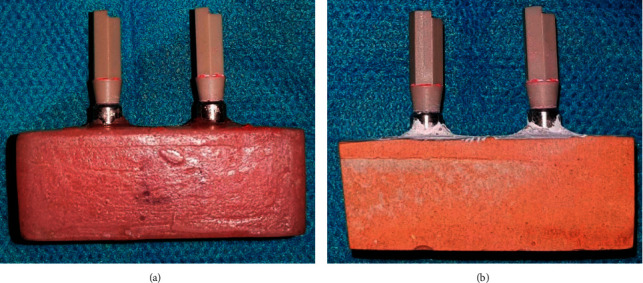
(a) The fabricated model. (b) The prepared casts.

**Figure 5:**
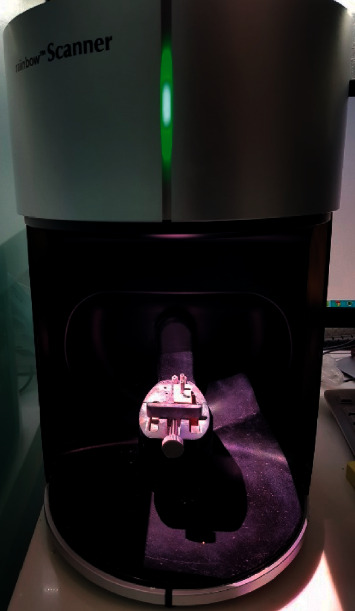
Laboratory scanner.

**Figure 6:**
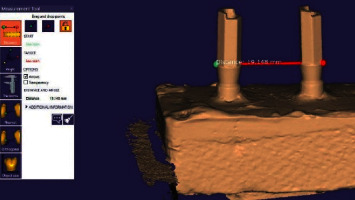
The designed image in CAD software.

The PEEK scan bodies screw-retained on the main model in each group were separated after the setting of the plaster and were exactly screw-retained on the same part of counterpart analogs on the cast in the clockwise direction at 5 Ncm torque (Figures 4(a) and 4(b)). The casts were scanned similar to the initial sample by a laboratory scanner (Figure 5) using the CAD software (Figure 6).

### 2.5. Measurements

CAD software version 2015.07 (Dental CAD, Exocad GmbH, Darmstadt, Germany) was used for all measurements. The distance between the outer hex in the prepared virtual models was digitally measured by the same operator using the measurement tool in CAD software through marking the scan bodies. Measurements were made at the points where three lines on the outer hex of each scanbody meet. The distance was calculated in millimeters. The mean of four measurements on each sample was reported considering the interclass correlation coefficient (ICC). All measurements were made by the same operator (one of the authors) using Exocad software.

The intraclass correlation coefficient was applied to evaluate the agreement between the different measurements of an observer at different times, which was reported at >97% in the current study. Consequently, the mean of four measurements was used to report the distance. The numbers obtained for each group were recorded and compared with each other and also with the distance in the main model.

### 2.6. Statistical Analysis

The data were analyzed by descriptive statistics (mean ± SD) and the Kruskal–Wallis test for comparing the three groups using SPSS 17. *P* < 0.05 was considered significant.

### 2.7. Outcome of Study

The present study aimed to evaluate the accuracy of different splint methods for implant impressions made at different times. The obtained results demonstrated that sectioning the splint after 17 minutes and 24 hours did not lead to a significant difference in the accuracy of impression. Then, based on the obtained results, the study hypothesis is accepted.

## 3. Results

The mean distance measured in the impression immediately after the setting time was 19.14 ± 0.029 mm. The one-sample *t*-test demonstrated that the difference between this impression technique and the real measure was significant (*P*=0.022). The mean distances measured in impression after 17 min and 24 h with sectioning and reconnecting the splint were 19 ± 15.039 and 19.159 ± 0.033 mm, respectively (Table 1). The latter measures were not significantly different from the real value (*P*=0.617 and *P*=0.0403). A comparison of numbers resulting from measurements made in the three groups did not reveal a significant difference (Table 2).

## 4. Discussion

With growth in impression methods and impression materials, a prosthodontist should select the suitable impression method and impression material with its good technical information for a specific case. While a variety of procedures for making impressions of implant-retained prosthesis have been reported, each one shows their weaknesses.

An accurate impression method is important, since the inappropriate method may lead to mechanical or biological problems that finally cause to the failure of implant restoration. San et al. assessed the linear dimensional accuracy of implant impressions utilizing four silicone impression materials and different impression methods. Their results showed that both addition and condensation silicones may certify satisfactory accuracy with either the closed tray or open tray method for implant impressions [22].

In this in vitro study, the accuracy of different splint methods for implant impressions at different times was evaluated. Taking one-step putty reline impression through sectioning and reconnecting after 17 minutes and 24 hours was not significantly different from the real value in terms of accuracy. Furthermore, the distances measured in the three splinting techniques were not significantly different.

Cabral et al. concluded that direct impression techniques with squared transfer copings with acrylic resin splints are better than indirect and nonsplinted methods [23]. Stimmelmayr et al. observed a significant difference between the nonsplinted copings group and splinted copings with acrylic resin. The group with splinting had the lowest standard deviation [17]. Assif et al. reported that the accuracy of impression increased when the impression copings were splinted before taking an impression in the direct impression-taking method. They justified it by preventing the separate movement of impression copings during the impression-taking process by rigid splinting of components to each other [24].

Although most previous studies have recommended splinting the internal connections of implants, some others have reported different views. Baig demonstrated that the two impression methods with and without splinting were not different in terms of impression accuracy [25]. Hsu et al. did not observe a significant difference in the measurement accuracy of joined and separate square impression copings [26]. An autopolymerized acrylic resin (GC pattern resin LS) pattern was used as the splinting material for joining the impression copings in the present study.

To overcome the polymerization problems during the splinting process of impression copings, in the current study, sufficient time was allowed to elapse in groups two and three before taking an impression (17 minutes and 24 hours) to complete polymerization. This time resulted in high accuracy of the impression. Although measurement accuracy immediately after the setting time (4 minutes) was significantly different from the real sample, the accuracy 17 minutes and 24 hours after the splinting was not similar to the real sample.

According to Mojon et al., the highest rate of shrinkage of the resin pattern (80%) (DuraLay, Reliance Dental Mfg. Co., Worth, III) occurred in the first 17 minutes after mixing the powder and liquid. During 24 hours, 45–75% of double bonds reacted, and 97% of the final shrinkage took place. After 24 hours, measurable changes do not happen in terms of polymerization shrinkage [18]. In addition, Gibbs et al. reported 5.72% and 5.07% shrinkage rates in GC Pattern resin and DuraLay, respectively [12].

Lee and Cho in 2011 evaluated the impact of splinting materials and techniques on the accuracy of impressions. They found that taking an impression of splinted components using autopolymerized resin with sufficient time for polymerization (24 hours) and sectioning for compensating the shrinkage is the most accurate splint technique, and the technique with splinting and taking an impression with plaster has an acceptable accuracy [20].

Deogade reported that cutting the acrylic bars 24 hours after polymerization and joining them again in the oral cavity provides enough time for impression copings for resin shrinkage. As a result, the highest accuracy is obtained for taking impressions from several implants for fabricating a one-piece framework [27].

Dumbrigue et al. proposed a more rapid method to reduce the shrinkage of acrylic resin used for splinting the transfer copings by using prefabricated bars [28].

In the present study, the sectioned splint method was applied in two groups because this technique is of particular importance in impression accuracy. Cabral and Guedes concluded that splinting is more accurate in the case of sectioning and reconnecting. However, splinting without sectioning exhibited the lowest accuracy [23]. In addition to time, the splinting technique affects the distortion and shrinkage of polymerization. Sectioning and reconnecting the splint might prevent movements, shrinkage of splint materials, and fractures [19].

Papaspyridakos et al. in 2011 found that splinting with acrylic resin coupled with sectioning and reconnecting the splint leads to master casts that are more accurate than the nonsplint method [29]. Tarib et al. indicated that impression through the direct method and splinting with sectioning just before the final impression has the highest accuracy [30].

Öngül et al. reported that the splinting technique utilizing autopolymerized acrylic resin was more accurate than other methods, and the sectioned bars exhibited a considerable difference from the main model [31], which is different from the current study. This controversy could be attributed to the prefabricated acrylic bars made 24 hours in advance in the study above.

We applied the splinting technique in all the three groups, and acceptable accuracy was observed compared to the main sample. In the two methods, which were coupled over time, impression accuracy did not significantly differ from the real sample. On the other hand, a significant difference with the real sample was demonstrated in the group in which the impression was taken immediately after the setting time, which was not yet acceptable.

In a recent study, Bacchi et al. assessed the effect of framework material and vertical misfit on stress formed in an implant-supported partial prosthesis under load application. Their results showed that when the vertical misfit increased, the stress values also increased in all of the prosthetic systems and periimplant bone tissues. The framework material and vertical misfit level revealed a related impact on the stresses for all of the tested structures [32].

The discrepancy in the outcomes of different reports might be attributed to the varying amounts of splint material that affect the shrinkage of resin during polymerization. Furthermore, the dimensional changes of utilized plaster during fabricating the cast are important. The volume of plaster and dilation due to the setting reaction can influence the impression accuracy. The material volume was unified in the present study because an index was made for splint material, plaster, and impression material.

## 5. Limitations

One of the reasons for controversial findings could be variable impression materials rather than the differences in the impression technique, and it should be noted that we utilized putty and wash impression material (Kettenbach GmbH and Co., KG, Panasil, Germany). In addition, the splint material could be another reason for the variable results.

The studies mentioned above used different materials for splinting. Lee and Cho found that making an impression of splinted impression copings using autopolymerized resin can compensate for polymerization shrinkage, and the splint technique of making an impression with plaster can improve the accuracy of the master cast [20]. In the study conducted by Öngül et al., the splint fabricated with acrylic resin was more accurate than light-cured composites [31].

One of the limitations of the current study, the correction of which might improve the accuracy of the impression-taking procedure, was the elastic putty nature of splint indices. A similar and more accurate splint material volume can be achieved by making the indices more rigid. Moreover, further clinical studies can better clarify the impact of oral cavity conditions on the materials used for splinting.

## 6. Conclusion

The present study aimed to evaluate the accuracy of different splint methods for implant impressions made at different times. The obtained results demonstrated that sectioning the splint after 17 minutes and 24 hours did not lead to a significant difference in the accuracy of impression. Therefore, dentists can make an impression after 17 minutes to reduce chair time and make an optimal treatment with less complications.

## Figures and Tables

**Table 1:** Comparison of impression accuracy.

Splinted, impression after the setting time (4 min)	*N*	Mean	Standard deviation	One-sample *t*-test (value = 19.164)			
				Mean difference	95% confidence interval for the difference		*P* value
					Lower bound	Upper bound	
Distance	12	19.1417	0.02907	−0.0223	−0.0408	−0.0039	0.022

17 minutes, cutting and splinting again	*N*	Mean	Standard deviation	One-sample *t*-test (value = 19.164)			
				Mean difference	95% confidence interval for the difference		*P* value
					Lower bound	Upper bound	

Distance	12	19.1541	0.03949	−0.00992	−0.035	0.0152	0.403

24 hours, cutting and splinting again	*N*	Mean	Standard deviation	One-sample *t*-test (value = 19.164)			
				Mean difference	95% confidence interval for the difference		*P* value
					Lower bound	Upper bound	

Distance	12	19.1590	0.03369	−0.00500	−0.0264	0.0164	0.617

**Table 2:** Comparison of impression accuracy between the three groups and real value.

	*N*	Measured distance		Difference from the real value	
		Mean	Standard deviation	Mean	Standard deviation
Splinted, impression after the setting time (4 min)	12	19.1417	0.0291	−0.0223	0.0291
17 minutes, cutting and splinting again	12	19.1541	0.0395	−0.0099	0.0395
24 hours, cutting and splinting again	12	19.1590	0.0337	−0.0050	0.0337

*P* value			0.453		

## Data Availability

The data used and/or analyzed during the current study are available from the corresponding author.
